# Rural Health Disparities: Contemporary Solutions for Persistent Rural Public Health Challenges

**DOI:** 10.5888/pcd22.250202

**Published:** 2025-06-19

**Authors:** Kevin A. Matthews, Katie S. Spears, Charkarra Anderson-Lewis

**Affiliations:** 1 Office of Rural Health, Centers for Disease Control and Prevention, Atlanta, Georgia; 2 Oak Ridge Institute for Science and Education, Oak Ridge, Tennessee; 3 University of Southern Mississippi, Hattiesburg

Rural communities in the United States face significant public health challenges, including limited access to physical and mental health care, inadequate health care infrastructure, low rates of participation in leisure physical activities, and restricted availability of healthy food. These challenges underscore the growing divide between rural and urban areas, calling for solutions beyond traditional methods. This collection of articles in *Preventing Chronic Disease* (PCD), “Rural Health Disparities: Contemporary Solutions for Persistent Rural Public Health Challenges,” emphasizes building trust in developing effective public health strategies for rural regions. Sustainable progress relies on community-driven approaches that recognize the strengths of these regions. Enhancing trust and collaboration requires culturally sensitive communication and engaging with local communities while avoiding harmful stereotypes.

Public health professionals must prioritize rural perspectives and commit to ongoing collaboration. This collection illustrates the crucial role of trust in improving public health in rural America. As suggested in the title of this collection, public health challenges persist in rural America. These challenges lead to higher rates of chronic health conditions in many rural communities compared with urban communities, disparities that have been growing since the mid-20th century.

## Overview of the PCD Collection

The 9 articles in this collection highlight 2 overarching themes: type of chronic condition concerning the well-being of people living in rural areas and type of inquiry (programmatic vs quantitative/analytic) addressed by the authors. By type of chronic condition, 3 articles addressed obesity linked to food and leisure physical activity (1–3); 3 articles focused on diabetes (4–6); and 3 articles examined cancer (7–9). Six of these articles adopted a programmatic approach, describing and outlining programs and highlighting the need to customize initiatives for the needs of each rural community: 2 articles on obesity (1,2), 2 articles on diabetes (4,5), and 2 articles on cancer (7,8). The remaining 3 articles adopted a quantitative and analytic approach: 1 article on obesity (3), 1 article on diabetes (6), and 1 article on cancer (9).

For programmatic approaches to obesity, Gallagher et al (1) focused on identifying the essential elements of a physical activity program for men in rural regions. This article examined the importance that men living in rural areas place on physical activity for their health and their enthusiasm for the program. Smarsh et al (2) highlighted the importance of public transit availability, demand-responsive transportation, and demand-responsive transportation services that connect food retail sites, such as farmers markets and supermarkets, in transit planning.

For programmatic approaches to diabetes, Saiki et al (4) explored the enrollment of people with prediabetes in a lifestyle change program, uncovering motivators and barriers to participation in initiatives similar to the National Diabetes Prevention Program lifestyle change program. Sastre et al (5) discussed a produce prescription initiative designed for low-income residents who have diabetes and lack health insurance. Their results showed an increase in fruit and vegetable intake, accompanied by a decrease in hemoglobin A_1c_ levels, underscoring the importance of formative and process evaluations in determining the relevance and effectiveness of produce prescription programs.

For programmatic approaches to cancer, Allen et al (7) explored cancer prevention activities and collaboration facilitators among informal interagency networks in rural areas. Kava et al (8) performed a scoping review of colorectal cancer screening interventions in rural areas. Many interventions reviewed were multicomponent, demonstrating that colorectal cancer screening interventions may be improved by using theory-based approaches, assessing costs, expanding populations covered, and tailoring to community needs.

Three articles adopted an analytic approach. Crouch et al (3) evaluated food security, physical inactivity, and rates of overweight or obesity in children and adolescents living in rural and urban areas. It offered essential insights for developing strategies to promote healthy weight and prevent obesity, enhancing public health efforts and shaping effective dietary and exercise programs. Khavjou et al (6) found significant geographic disparities in diabetes prevalence among adults between rural and urban areas in 19 states. Schulz et al (9) used spatial analysis methods to investigate sociodemographic factors that contribute to breast cancer mortality rates. Their findings can help guide targeted resource allocation and public health strategies.

## The Unique Characteristics of Rural Communities

It is important to acknowledge that rural communities are not monolithic; they differ across various factors that shape public health priorities and outcomes. Each possesses a unique combination of characteristics, including socioeconomic status, racial and ethnic composition, health care access, environmental conditions, industry presence, and historical context. For example, the public health challenges of a rural agricultural region in the Midwest may differ from those of a former mining town in Appalachia, a frontier community in the West, or a beachfront recreational area on the East Coast. Rural communities often face disparities in key social determinants of health, such as education and housing. A low or declining tax base worsens these challenges, and they were further intensified by the effects of the COVID-19 pandemic. Rural populations are aging rapidly, driven by aging in place, retirement migration, and the out-migration of younger residents. This trend contributes to higher rates of chronic health conditions and increased demand for health services (10). These differences affect the health needs, challenges, and resources of each community. 

Addressing persistent rural public health challenges requires a multifaceted strategy incorporating contemporary solutions. This PCD collection explores several approaches, including multisectoral collaborations, spatial analysis for resource allocation, community engagement, tailored and appropriately designed programs, engaging trusted individuals, and delivering interventions through mobile applications. However, we note that the original Call for Papers for this collection marked an important shift. “Rural public health” is gaining recognition in the peer-reviewed literature, distinguishing it from the traditionally health care–focused use of the term “rural health.” The disciplinary origins of public health initially concentrated on the epidemiological challenges associated with high-density (urban) populations, such as sanitation and infectious diseases (11). Over time, its focus broadened to include rural communities, prompted by the rapid increase in chronic diseases during the mid-20th century. Currently, scientists in the peer-reviewed literature most often interpret the term “rural health” narrowly, focusing on treatment, access to care, and health care systems; however, growing interest in “rural public health” as a complementary field of inquiry presents an opportunity to integrate these perspectives so that rural health is more fully understood to include both health care and public health dimensions and their intersection.

## Rural–Urban Comparisons

When developing interventions or policies aimed at improving the health of rural populations, it is important to critically examine the usefulness of making only rural–urban health comparisons. Comparing health outcomes between rural and urban areas can be problematic because it oversimplifies the issue. While such comparisons highlight rural–urban differences, they may not provide actionable insights into which interventions will be most effective in rural America. Also, framing rural–urban differences as disparities is a deficits-focused approach, which may suggest that urban areas are somehow better than rural areas. We need to reframe our perspective: rural settings possess unique strengths and challenges that affect the population health of rural communities (12). Another symptom of the rural–urban dichotomy is the “us-versus-them” mindset, which portrays rural populations as unhealthy and needing saving, while casting urban populations as the ideal — overlooking the reality that rural and urban communities are mutually interdependent. Moreover, policies based on broad rural–urban comparisons might not be effective. A better approach may be to prioritize within-group comparisons — examining differences among rural populations, such as across different rural areas, between population groups within the same rural area, or among similar groups across multiple rural areas — to identify communities with better health outcomes and extract insights that are more relevant and actionable than those from traditional between-group (rural vs urban) comparisons.

## Trust-Building, Partnerships, and Collaboration

It is important that public health workers not lose trust before they have the opportunity to build it. Trust can be damaged by how people outside their communities portray rural communities. Language that pathologizes rural life or assumes rural identity is inherently linked to poor health is counterproductive. For examples, terms like “rural mortality penalty” (13–16) can imply that adverse outcomes are due to personal failings rather than structural shortcomings, such as limited health care access, poor infrastructure, or economic disadvantage. Such framing can lead to reinforcing blame rather than promoting understanding. Instead, public health professionals can build lasting trust by using respectful and inclusive language and including and honoring rural voices (17). Developing public health communications that rely on a community’s leaders or subject matter experts may resonate with rural people more than deficit-focused, prescriptive, or condescending language from researchers, scientists, or government officials who are not community members (17). Interpersonal connections and community-engaged approaches can be more effective in health communication than a top-down approach in rural areas with low-trust contexts.

## Rural Public Health Training

A key approach to developing trust and addressing rural public health challenges is building a robust and culturally competent workforce. This involves creating a talent pipeline through targeted rural public health training for students, early-career professionals, and seasoned public health practitioners transitioning to serve rural communities. Key content areas would include understanding rural determinants of health, addressing health disparities, designing tailored interventions, applying rural research methods, navigating data challenges, being aware of the intersection of public health and health care in rural settings, and evaluating programs and policies in rural settings. In addition to developing a rural public health training curriculum, efforts to build a robust rural public health infrastructure could also encompass fellowships and other experiential learning opportunities. Recruiting students, faculty, and public health professionals from rural areas — or those with significant lived rural experience — can also enhance workforce capacity development to respond effectively to rural health challenges and foster trust and alignment with rural communities.

## Final Thoughts

We noted that the original Call for Papers reflects a growing recognition in the peer-reviewed literature that rural health must be understood not solely through the lens of health care delivery, but as a broader field that includes rural public health and the intersections between public health systems and the health care infrastructure in rural America.

Two themes emerged from the articles selected for this collection: chronic health conditions in rural communities and the type of inquiry used, each theme signifying the need for culturally tailored messaging and public health initiatives for each rural community. Effective public health in rural areas requires respectful, collaborative engagement, rather than top-down solutions that ignore local context, and public health professionals must be self-reflective to avoid reinforcing harmful stereotypes about people living in rural areas. Additionally, improving health outcomes in rural communities necessitates more than comparing them to urban counterparts — it demands tailored, respectful, and collaborative approaches that honor rural experiences and expertise. Tailoring public health initiatives to the unique context of each rural community is more effective than a one-size-fits-all approach. Trusted local partners, such as cooperative extension services and health departments, can help engage communities by building on rural strengths such as collaboration, social cohesion, and local pride (18). Public health professionals can build stronger partnerships and promote sustainable change by fostering trust, empowering local leaders, and avoiding reductive language.

Rural public health aims to support conditions that enable all people — regardless of geography or circumstance — to achieve their optimal health. This approach requires more than top-down interventions; it demands sustained, community-informed efforts that center rural voices and lived experience in designing, implementing, and evaluating public health strategies.
